# The Health Literacy of U.S. Immigrant Adolescents: A Neglected Research Priority in a Changing World

**DOI:** 10.3390/ijerph15102108

**Published:** 2018-09-25

**Authors:** Maricel G. Santos, Anu L. Gorukanti, Lina M. Jurkunas, Margaret A. Handley

**Affiliations:** 1 Department of English, San Francisco State University, San Francisco, CA 94132, USA; 2 Department of Pediatrics, Stanford University, Stanford, CA 94305, USA; agorukan@stanford.edu; 3 American Language Institute, San Francisco State University, San Francisco, CA 94132, USA; ljurkuna@mail.sfsu.edu; 4 Department of Epidemiology & Biostatistics, University of California, San Francisco, CA 94158, USA; Margaret.Handley@ucsf.edu; 5 Department of Medicine Division of General Internal Medicine, Zuckerberg San Francisco General Hospital, San Francisco, CA 94110, USA

**Keywords:** immigrant adolescents, health literacy, immigrant identity, adaptation

## Abstract

Immigrant adolescents are the fastest-growing sector among U.S. youth, but they receive little attention in health literacy research. Immigrant adolescents are a diverse population tasked with mastering new literacies while also navigating new social systems. Many immigrant adolescents serve as important linguistic and cultural resources in their families and local communities, and yet their contributions (and struggles) as new navigators of our health care system remain invisible. In this commentary article, we argue that health literacy researchers need to devote more attention to immigrant adolescents and the pathways by which they learn new language and literacy skills while also developing their own health habits and behaviors. We contend that the study of immigrant adolescents provides a critical window into health literacy as a socially and historically situated practice, specifically how immigrant adolescents’ transnational experiences shape their learning of new health literacy practices. With a coordinated interdisciplinary research agenda on immigrant adolescents, the health literacy field will expand its empirical base for what becoming “health literate” looks like in today’s globalizing world.

## 1. Introduction: Becoming “Health Literate” in a Globalizing World

Over the past decade, the number of U.S. children with at least one immigrant parent has been on the rise, increasing from 19% to 26% of all children in the U.S. [1]. The large concentration of English language learners who entered U.S. schools in preschool or elementary school earlier this decade are now moving into higher grades, generating marked increases in immigrant youth in U.S. secondary schools [2]. Currently, nearly three-quarters of immigrant youth of ages 12 to 17 are U.S.-born [3]. The recent “diaspora of teenagers” makes up two-fifths of first-generation children entering the U.S. [4]. The socioeconomic opportunities available to immigrant adolescents are also shifting in today’s information-driven economy, a trend that does not bode well for the 52% (3.1 million) of immigrant adolescents living in low-income households [5]. For convenience and ease of expression, we use the term “immigrant adolescent” to refer to an adolescent from an immigrant family, i.e., lives in a home with at least one immigrant parent, regardless of country of birth, including adolescents in refugee families. However, in this paper, we address experiences that may distinguish foreign-born from U.S.-born youth, and highlight unique health literacy opportunities of children in refugee families.

Despite the fact that immigrant adolescents are the fastest-growing sector of the U.S. immigrant population [3], they remain invisible [6] in health literacy research. With rare exceptions, there has been insufficient attention to the health literacy capacities of adolescents, much less that of immigrant adolescents [7,8,9,10]. This research chasm is especially troubling since the efficacy of many health care programs and services may depend on how this cohort of immigrant adolescents fares as a whole, with lasting effects into adulthood.

Despite widespread consensus that health literacy occurs in a social context, the field has yet to fully examine the social and historical circumstances that are dynamically shaping the development of new health literacy skills and practices in immigrant communities. Today’s immigrant adolescents are coming of age in a rapidly changing world: unprecedented migratory, technological, and institutional forces are structuring their childhoods, while at the same time, their demography, experiences, and choices are altering the immigration narrative. 

In response to this empirical gap, our commentary focuses on how the lived, everyday transnational experiences of U.S. immigrant adolescents provide a meaningful context for the emergence of health literacy competence. To what extent do immigrant adolescents encounter opportunities to develop their health literacy in their everyday lives? What sources of information and literacy tools are immigrant adolescents motivated to use, and what are the consequences of these navigational practices for their health literacy development? Drawing on the terminology in Donald Nutbeam’s health literacy framework [11], we must ask, to what extent are these everyday interactions and choices laying the groundwork for the acquisition of functional, communicative, and critical levels of health literacy in the immigrant adolescent population? Answers to these questions require a coordinated, interdisciplinary research agenda. This basic claim will be repeated throughout our commentary, as we discuss several distinct areas of research on the immigrant adolescent world where this claim could be explored.

This commentary has three goals: (1) to make a case for such a research agenda on immigrant adolescent health literacy; (2) to highlight the kinds of research questions on immigrant adolescent health literacy that productively build on existing theories and research on literacy as a social practice, adolescent literacy, and immigrant adolescent identity; and (3) to underscore the need for interdisciplinary collaboration in support of improved health literacy outcomes among immigrant adolescents. The focus of this commentary is on U.S. immigrant adolescents, but we anticipate that our call for research will resonate with practitioners in other parts of the world who already recognize the need to pay more attention to the needs of immigrant adolescent populations.

For this commentary, we undertook a targeted multidisciplinary approach to explore promising areas of research on immigrant adolescent health literacy. As literacy educators, we were compelled to revisit a seminal review paper “Re-Framing Adolescent Literacy Research for New Times: Studying Youth as a Resource” by Elizabeth Moje in which the author asserts that the “lack of attention to youth literacy... points to unstated assumptions among theorists and policy-makers alike that nothing occurs in the literacy development of youth, that no learning about literacy occurs as youth make use of literacy tools to navigate, resist, construct, and reconstruct popular, academic, and work cultures” [12] (p. 211), an assertion that may also partly explain why relatively little attention has been paid to adolescent health literacy in the health literacy field. Building on Moje’s insights into adolescent literacy, youth culture, and youth literacies, we used JSTOR and Linguistics & Language Behavior Abstracts to look for qualitative studies (largely ethnographic case studies) that provide rich descriptions of immigrant adolescents’ everyday literacy practices. We focused on case studies published after the 2010 U.S. Census as we wanted studies that reflected recent U.S. demographic and diasporic trends in the immigrant adolescent population, although we indicate when we draw on relevant literature published in other countries. This exploration enabled us to imagine the range of literacy experiences, resources, and interactions that could be investigated with an immigrant adolescent health literacy agenda. We also explored the health literacy literature via PubMed using the following search terms: adolescent (youth) health, health literacy, immigrant/refugee, and United States. Because of our interest in health literacy as a situated practice, we also specifically targeted articles that did not adhere to strictly functional views on health literacy. 

While the relationship between health literacy and health outcomes has been productively modeled [8,13,14,15], these models have yet to be fully explored with respect to adolescent health literacy (with Manganello [7] an important exception) or immigrant adolescent health literacy specifically. Thus, for this commentary, we have drawn upon several review titles in public health that expressly aimed to advance “adolescent health literacy” as a unique phenomenon [7,8,9,10]. Perry [8] conducted an integrative review of the health literacy literature, with a special focus on children with chronic illness, concluding that there remains a scarcity of literature focusing on health literacy instruments and intervention in adolescents. A comprehensive review conducted by Sanders et al. [8] emphasized the significant impact of poor literacy on poor health literacy, arguing for new measures of childhood literacy that take into account the developmental context of child health. The work of Manganello [7] and Higgins et al. [10] were particularly helpful because the authors applied socio-ecological models to explore how adolescent health literacy is linked to various social and environmental factors (peer networks, family characteristics, media, schools, and the health care system). Also significant is Fairbrother, Curtis, and Goyder’s study of child health literacy in the U.K., which embraced the social practices view in ways that most directly align with our own assumptions, but focused on younger (pre-adolescent) children ages 9–10 [16]. Our hope in this commentary is that, with more research on the social contexts in which immigrant adolescents are developing their health literacy competence, our field will be better poised to empirically validate the pathways that link immigrant adolescent health literacy to immigrant adolescent health outcomes.

The following section explores reasons why immigrant adolescents have not received greater attention in the health literacy field. We then sketch some possible starting points for research as part of this proposed agenda on immigrant adolescent health literacy.

## 2. Explaining the Neglect of Immigrant Adolescents in Health Literacy Research

We suggest that the neglect of immigrant adolescents in health literacy research may stem from two enduring limitations in the conceptualization and study of health literacy. First, past health literacy research has focused nearly exclusively on adult populations. Research shows that many immigrant adolescents are able to acculturate more readily to life in the U.S. and gain proficiency in English ahead of their parents. Although the immigration literature abundantly supports the idea that the acculturation process is a function of a child’s development stage [17], this insight seems to have barely influenced thinking about the development of health literacy among immigrant populations across the life span.

Second, the health literacy field’s adherence to conceptualizations of health literacy as an individual’s ability to read and write in English likely perpetuates deficit perspectives on immigrant adolescents who are still learning English. The bias towards health literacy as a competence linked to one’s proficiency in English discounts the possibility that health literacy competence for immigrant adolescents may involve moving across languages and cultures—a translingual, transcultural capacity, which is regarded as a normative aspect of the migration and adaptation process [18,19]. We are not aware of any health literacy framework that fully accounts for this sociolinguistic reality in immigrant communities. 

## 3. Understanding Immigrant Adolescent Health Literacy as a Socially Situated Practice

A functional view of health literacy, one that emphasizes an individual’s individual capacity to read, comprehend, and use health information, is pervasive in health literacy research, as widely promoted by the Institute of Medicine [20]. We prefer to think of health literacy as a socially situated practice, in alignment with researchers whose scholarship is rooted in paradigmatic shifts associated with New Literacy Studies in literacy scholarship [21,22,23] and the “social turn” in applied linguistics [24]. To understand health literacy as a socially situated practice, the reader needs to consider what literacy is. A well-rooted convention in literacy teaching and research is the idea that literacy is a discrete set of skills that can be learned in a step-by-step fashion: learners must learn foundational skills, such as phonics (i.e., linking sounds with their correct letter combinations), or spelling, as well as higher-order information-processing skills for understanding a text’s purpose, audience, and message. The skills view tends to focus on literacy as an individual achievement, reflected in the learner’s ability to put autonomous skills to use in different contexts.

The *literacy as social practice* view recognizes a focus on skill-building is essential but not sufficient if we want to fully understand “the variety of literacy-related activities that individuals and communities engage with in their everyday lives or the range of meanings literacy has, depending on its social and cultural context of use” [25] (p. 24). The social practice view provides health literacy researchers with a framework for understanding how health literacy skills are shaped by the social contexts, purposes, and relationships within which reading, writing, math, or speaking skills are put to use.

Consider, for example, the context in which the basic task of making a doctor’s appointment takes place. An appointment could be scheduled for oneself or behalf of someone else. The physical act of making the appointment, and the resources required (e.g., access to the Internet, a phone, or a directory) vary depending on how the appointment is made (in person, by phone or online, or with or without language assistance). The kind of information required to make the appointment may vary based on whether the person is a new or established patient, and the kind of health information to be exchanged at the appointment (is it an acute health care issue, wellness checkup, or consultation?). The social view of health literacy is concerned with these local particularities as well as with any “standards” that dictate what one is “supposed” to do to complete health care tasks. For these reasons, the social view uses the term “literacy practices” to refer to both what people do with their language and literacy skills and how people understand, feel, and value those skills and behaviors.

Despite significant efforts to reconceptualize health literacy as a social phenomenon [11,26,27], the field has yet to shake its emphasis on health literacy “skills”, a bias that tends to reify the belief that health literacy outcomes should improve as people improve their language, literacy, and numeracy skills. Similarly, Pleasant has argued that the health literacy field, particularly in the development of health literacy measures, is struggling to differentiate health literacy as “the use of literacy skills in a health context” [28] (p. 1492) (the functional view) from health literacy as “a unique social construct that shares attributes with literacy” (p. 1491) (the social view). As we argue in this paper, we believe that a focus on immigrant adolescent health literacy provides an excellent platform for puzzling through these distinctions.

**Figure 1:**
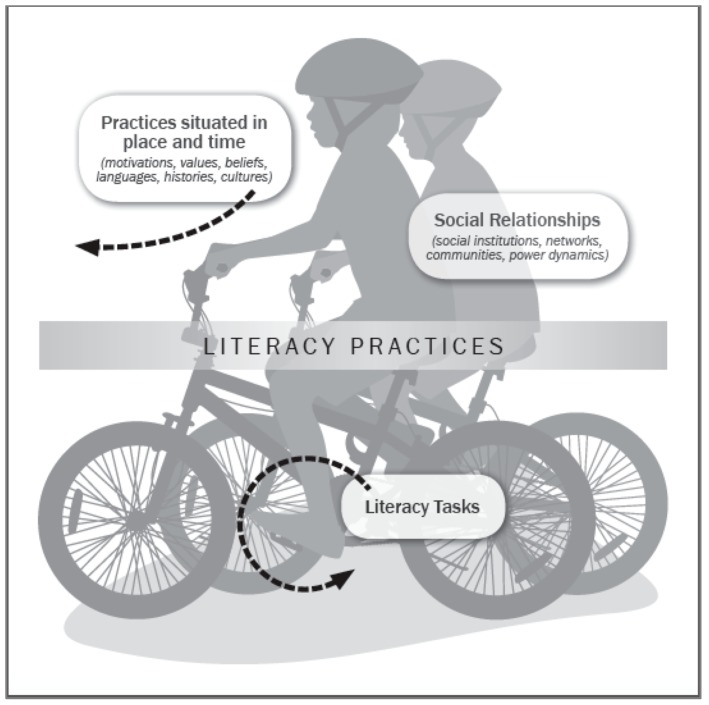
Understanding immigrant adolescent health literacy as socially situated practice: A bike-riding metaphor.

To further clarify our thinking about immigrant adolescent health literacy as a socially situated practice, we use a bike-riding metaphor (Figure 1), which takes inspiration from health literacy models described by the Institute of Medicine [20], Manganello [7], and Purcell-Gates [29]. Bike-riding requires the coordination of many working parts (gears, brakes, handle bars, and pedals). Each part has a unique purpose, but their contributions have little meaning apart from the whole. Similarly, we view an immigrant adolescents’ process of becoming “health literate” as an evolving coordination of many working parts (e.g., reading skills, math skills, form-filling skills, linguistic choices, digital tools, or interactions that involve any of these skills and tools). The significance of these parts cannot be accurately understood when apart from the social, cultural, and historical context in which immigrant children are growing up.

From a social perspective, the question—what is immigrant adolescent health literacy?—is tantamount to grappling with a more fundamental question, what does being “health literate” mean in a globalizing world with new opportunities for connectivity and communication? The terrains where adolescents end up riding their bikes represent the varied social, cultural, and institutional contexts—schools, our health care system, this period in immigration history, the political climate, and the neighborhoods where children grow up—that are dynamically shaping their early encounters with the health care system and creating new motivations or unique barriers.

As shown in Figure 1, the pedals represent “everyday literacy tasks”. Pedals set a bike in motion. Similarly, everyday tasks—such as interpreting a document for one’s parents or opting for water over a soft drink—may incentivize the application and perfecting of “little skills”, which may contribute to improvements in valued health literacy practices, such as effectively communicating with a health care professional or making good nutritional choices for oneself. The social practice view also holds promising implications for health literacy interventions, as we contemplate the kinds of everyday literacy tasks we could promote in the lives of immigrant adolescents, in and out of school, and across all levels of functional, interactive, and critical health literacy [11].

Just as learning to ride requires an ongoing “reading” of the road, learning to be “health literate” involves constant negotiation of one’s own skills and motivations in response to the demands of a particular environment. A social practice view on health literacy is fascinated by the kinds of adjustments people make (the tools they use, the people they seek out, the decisions they make, and the curiosities they act on) as they carry out health care tasks. A social practice view is not so preoccupied with evaluating whether immigrant adolescents make a “good” or “bad” adjustment but rather on richly describing what the coordination of “little” adjustments can look like.

Figure 1 depicts two children riding together, illustrating that the communal experience (bike-riding with friends) is often what gives lasting meaning to the experience of bike-riding for many children. Similarly, the social practice view privileges ways that immigrant adolescent health literacy cannot be understood apart from the child’s membership in social networks, her real and desired connections with other people who are also endeavoring to make sense of what it means to be healthy. In this regard, the social view is consonant with views on health literacy as an attribute of social systems, as articulated by Papen [27], Nutbeam [11], and the Calgary Charter on Health Literacy [30]. This emphasis on the socially interdependent nature of health literacy allows for the possibility that immigrant adolescent health literacy is a function of the child’s competence and practices of those around them (global and local peers, teachers/coaches, parents, health professionals, and other adults charged with their care and supervision).

Lastly, Figure 1 depicts the adolescents wearing helmets, required safety gear for reducing the risk of serious or fatal head injuries. To extend our bike-riding metaphor, gaining health literacy competence as an adolescent requires learning to recognize the link between risk-preventive behaviors and positive health outcomes. At the same time, the fact that over 87% of U.S. adolescents ages 13 to 18 years old report rarely or never wearing a helmet [31] is a sobering reminder that many adolescents may engage in behaviors that place them at higher health risk. The social view on immigrant adolescent health literacy is keenly interested in richly describing the adolescents’ interactions with health risk messages and understanding how these interactions shape or do not impinge on their risk perceptions and health behaviors; in other words, from the social view, risk comprehension, for many immigrant adolescents, should be viewed as a process of meaning-making, an active grappling with perceptions of risk and risk messages.

We suggest that a research agenda on immigrant adolescent health literacy requires that we examine immigrant adolescents’ health care experiences from the perspective of the adolescents themselves, allowing them to articulate their own stories and understandings, and not rely only on adult-informed perspectives to operationalize what health literacy competence should look like. In this commentary, we aim to highlight several promising research domains where it would be beneficial to explore decision-making processes undergirding health care practices, including the risky ones that immigrant adolescents engage in. We are excited about new lines of health literacy research as we anticipate that immigrant adolescents have a lot to teach us about how all of us learn complex health literacy processes over time and diverse contexts.

## 4. Promising Research Directions on Immigrant Adolescent Health Literacy

This section addresses several promising areas of research on immigrant adolescent health literacy, with an emphasis on socially situated views on health literacy. Readers are encouraged to view these research directions as starting points for interdisciplinary deliberation and partnership-formation, rather than a comprehensive research agenda on immigrant adolescent health literacy. As suggested with our bike-riding metaphor, we are particularly interested in exploring everyday opportunities in the lives of immigrant adolescents that motivate “pedaling forward”, in other words, that set in motion the learning of health literacy practices. 

### 4.1. How Do Linguistic Brokering Experiences Create the Conditions for Learning New Health Literacy Practices?

Research has shown that immigrant children play an active role in their family’s adaptation and integration processes [32]. Immigrant adolescents, whose English proficiency readily starts to surpass that of other adults in their household, often experience “role-reversal” when they must take on adult-responsibilities, as translators, interpreters, and mediators in institutional settings on behalf their family [33]. Studies have shown that, for many immigrant adolescents, linguistic brokering (the term used to describe these acts of translation and interpreting in immigrant families) is not viewed as a burden but normative, even a source of pride and a means to “protect” parents’ dignity in social contexts; parents often describe these encounters as opportunities to reinforce their children’s bicultural identity [34,35]. We acknowledge the serious legal and ethical concerns, as well as the potential health risks, when immigrant adolescents are asked to take on adult responsibilities in health care (e.g., violations of a family member’s confidentiality, mistranslations, and emotional stress on the child) [36]. At the same time, a social practices perspective provides a valuable point of entry for exploring what is often viewed as “taboo discursive terrain” [36] (p. 386). By examining how immigrant adolescents make sense of spoken and written health information, and navigate gaps in communication, across languages, and lines of authority, we are likely to gain insight into the conditions which foster the development of interactive and critical health literacy. 

Faustich Orellana et al. provide compelling evidence that linguistic brokering activates a range of linguistic and pragmatic skills that arguably correspond to functional, interactive, and critical levels of health literacy. For example, Faustich Orellana et al. highlight the experiences of “Sammy”, a 15-year old Latino adolescent, who “took charge for himself and his mother during his own hand surgery, including researching information about the surgery on the Internet beforehand” [34] (p. 517). Faustich Orellana et al. also report on the case of “Lucila” who exhibited a keen awareness of the sociopolitical complexities of having to translate for her mother when composing a formal letter of complaint against the family’s caseworker:

I remember that day and I remember the tension I felt as I listened to my mom angrily complain about the lady, and the pressure I felt to translate “properly”. I didn’t know what to say. I wanted the complaint to sound like it came from a grown-up, my mother, but I also wanted to stress how rude (the lady) was, writing that she was very impatient with our situation and that my mom felt very uncomfortable with her and that it was really hard for her to express herself and to understand the lady.

[34] (p. 519)

Faustich Orellana et al. credit Lucila for her “willingness to step into the ‘adult’ role” [34] (p. 519) and her persistence in finding the right language to convey her mother’s complaint in English. These examples suggest that immigrant adolescents’ experiences as linguistic brokers may reveal an emerging critical health literacy [37], particularly evident in the case of Lucila whose literacy practices (translating from oral colloquial Spanish to written formal English, composing a complaint letter in English) were motivated by a desire to assert her own voice and claim a sense of authority on behalf of her mother (by the way, after submitting Lucila’s complaint letter, her mother was assigned a new case worker). In a study of children’s linguistic brokering in the context of health insurance, Martinez et al. [38] found that immigrant children were able to figure out how various aspects of the health insurance system by “watching and supporting” their families as part of enrollment and management processes, even if the children were not included in any insurance-related decisions. For example, by assisting their parents to figure out the requisite “papers” or “cards” to present, or even which “line” to stand in at social service offices, the children seemed to gain important insight into the complexities of health insurance options based on family immigration status.

Linguistic brokering provides compelling insight into the ways that immigrant adolescents’ opportunities to develop health literacy may be uniquely tied to the sociolinguistic realities of transnational family life. While there is rich ethnographic data on linguistic brokering in education, sociology, and applied linguistics [33,34,35,38,39], we have yet to systematically study how linguistic brokering relates to the growth of immigrant adolescent health literacy. More detailed explorations of how immigrant adolescents learn to recognize and interpret the complex power dynamics of brokering encounters in health care and adjust their messaging, using available languages, modalities (spoken, written), and registers, seems vital if we want to understand how to effectively promote interactive and critical health literacy.

### 4.2. What Is the Relationship between Health Literacy and Identity Development among Immigrant Adolescents?

Identity development is widely recognized as the adolescent’s central task of cognitive, social, and psychological development [40]. The social practice view similarly argues that literacy development is a function of changes in an adolescents’ social roles and evolving sense of identity. As Dávila argues, “Attitudes toward reading, how often, how much, and what students read have much to do with their self and positional identities, as well as their aspirations in and beyond school” [4] (p. 642).

As applied linguists, we find Bonny Norton’s [41] concept of investment particularly useful for the study how immigrant adolescent identity may be related to changes in health literacy practices. Investment is defined as the degree to which a language learner ascribes social, cultural, and political value to the enterprise of learning new skills and competencies [42]. As Norton explains, learners invest in learning new literacy practices and trying out new literacy tools, “with the understanding that they will acquire a wider range of symbolic and material resources, which will in turn increase the value of their cultural capital. As the value of their cultural capital increases, so learners’ sense of themselves, their identities, and their opportunities for the future are re-evaluated” [42] (p. 87). An immigrant adolescent’s desire to learn English may reflect their investment in various social identities—as a high school student, child of immigrant parents, daughter, representative of the Latina culture, friend, and so forth. Ambivalence towards a language or literacy task, accordingly, may mark an immigrant adolescent’s struggle to communicate and be understood, and thus, a struggle to be recognized as a “good student” or a “helpful daughter”.

Identity research already suggests ways that immigrant adolescents may be invested in health literacy practices. As reflected in the previous section, research on linguistic brokering has found that immigrant adolescents are motivated, out of a desire to be helpful, to participate in a range of health care activities on their families’ behalf, such as translating and interpreting, making appointments by phone, filling prescriptions, completing medical documents, and researching treatment options on the Internet [34]. Participation as a linguistic broker may reflect an immigrant adolescent’s positive response to being given opportunities to showcase their skills across languages, as evident in the case of Lucila.

Dávila’s research [4] profiles a young Somali immigrant woman who exhibited strong investment in passing a driver’s education course: she needed to drive because she was tasked with the grocery shopping and preparation of home-cooked Somali meals for the family, a task that is clearly tied to her family’s health. This study points to possible gendered dimensions that propel health literacy development for immigrant female youth differently than for their male counterparts.

Rubinstein-Ávila [43] profiles a Dominican female teenager whose linguistic brokering for her mother during doctor’s appointments revealed a level of English proficiency that was largely invisible to her school teachers who regarded her as a “reticent” language learner. Children’s personal health experiences may motivate health literacy learning, as shown in Moje et al.’s study of a 15-year old boy whose prior personal history with childhood asthma and air pollution in Mexico City motivated him to study air-quality concepts in his U.S. high school science class and even investigate ecology as a career [44]. Moje et al. indicate that the boy’s teachers were unaware of the source of his personal investment in the environmental ecology unit.

Longitudinal studies of immigrant adolescents’ investment in health literacy practices would be able to investigate changes in health literacy competence are tied to their transnational identities as they transition from adolescence young adulthood and assume greater independence in their health care decisions. Investment research in the health literacy field also stands to make an important contribution to our understanding of the intersection of emotions (desire to learn) and literacy growth that guide adolescent immigrants’ self-regulation and decision-making in early health care experiences.

### 4.3. To What Extent Do Immigrant Adolescents Use Their Digital Skills and Online Networks for Learning New Health Literacy Practices?

Studies on adolescent health literacy and digital environment tend to rely on a functional view of health literacy with an emphasis on knowledge or skill acquisition, such as the honing of effective information seeking skills in online environments [45,46,47]. In the adolescent literacy field, while there have been numerous studies of their digital literacy practices [48,49], we need more research that systematically documents the communication skills, the social networks, the array of informational resources, and the motivations/attitudes that mediate immigrant adolescents’ early encounters with public health messages or the health care system.

We can draw upon a growing body of literacy studies that recognize access to the Internet as a major driver of immigrant adolescents’ social and cultural adaptation to life in a new country at a pivotal time of identify formation. Elias and Lemish contend that the digital lives of immigrant adolescents should not be simply viewed as “a playground” for adventures, pleasurable experimentation, and risk-taking but a critical, “safe ground” [50] (p. 548) where immigrant adolescents are motivated to seek out resources (e.g., online news about current events in the home country; local youth activities) and explore social networks that reinforce their sense of belonging to the home country and the new country.

Earlier in our commentary, we cited the case of “Sammy” who searched the Internet to find medical information for himself and his mother about an upcoming surgery on his hand [34]. Similarly, Lam [51] has showed that immigrant youth routinely seek out online news resources in the U.S. and home country, a literacy practice, in the context of health care, would likely lead adolescents to a broader array of choices, with respect to language, content, and perspective, on a health care topic. This example suggests important research lines: In what ways do immigrant adolescents enable their families to “leapfrog” (Christina Zarcadoolas’s term) from the traditional world of print (where public health information in languages other than English may be limited) to the world of digital technologies and a dizzying amount of health information online? In what languages do immigrant adolescents search for health information, and to what extent does their knowledge of multiple languages prompt them to seek out information from a broader array of health information?

While this kind of seeking and sharing of online information seems ubiquitous in today’s digital world, how these digital literacy practices contribute to improvements in health literacy practices merits closer empirical investigation. As Manderino and Castek observe, “learners’ ability to use the Internet’s networking and knowledge-building resources is only as good as their skills in disciplinary inquiry: asking questions, constructing meaning from data, generating creative solutions, and reflecting on how to improve these solutions for different contexts” [52] (p. 79). “Disciplinary inquiry skills” here refers to the literacy practices that experts value when learning and problem-solving in their disciplines. This observation raises interesting conceptual and empirical questions regarding the relationship between digital literacies and immigrant adolescent health literacies. What exactly do “expert” digital literacy practices look like in health care contexts, and to what extent do immigrant adolescents’ digital literacy practices approximate those “expert” practices? 

Existing research also suggests that immigrant adolescents may cope with acculturative stress through participation in the digital world. For example, Gilhooly and Lee [53] examined the digital literacy practices of three Karen teenage brothers whose family left Burma and lived for several years in a Thai refugee camp before moving to the U.S. The teenagers learned to use a computer after arriving in the U.S. and, like Sammy in Faustich Orellana’s study [34], frequently used the Internet to find and share information, such as school immunization requirements. Perhaps more poignantly, the teenagers used their digital tools to create original music videos, short autobiographical films, and photo montages that communicated their political views on the political conflict in Burma, their experience in a Thai refugee camp, and their pride in Karen cultural symbols and traditions. The authors assert that these digital literacy practices were critical to the teenagers’ adaptive resilience: through their social media platforms, the boys were able to experience a sense of solidarity with distant Karen communities as well as forge new friendships with peers in the local Karen community. The boys were able to use their digital practices to forge what McLean [54] calls a “space for home” in the digital environment. As this example suggests, the significance of digital literacies for transnational adolescents seems to lie at the intersection of the adolescents’ desire for self-expression and connectivity with others, their access to digital tools and digital networks, and their response to immigration stress and adaptation.

The study of immigrant adolescent digital practices can reveal how literacy learning is motivated by children’s transnational knowledge and migration experiences. An example of adolescents’ creative expressions of their health concerns in the digital world is evident in The Bigger Picture Project [55], a collaboration between university researchers, medical practitioners, and community-based youth organizers, this initiative engages low-income and minority adolescents as youth poets in the production of video-based public service announcements (PSAs) (composed in the spoken-word genre) that are shared among youth via multiple social media platforms. Several of the videos highlight the adolescents’ hopes and fears about staying healthy in the face of broader social and environmental factors, including racism, poverty, and food insecurity. For example, in the public service announcement video entitled “Un sabor de casa” (translation: “A taste of home”, https://www.youtube.com/watch?v=yuhhxTj5od0), youth poet Monica Mendoza ties the pervasive consumption of soda among her peers and family to conflicts in her cultural identity: a nostalgia for home competing with feelings of resentment towards harmful TV advertising, as captured in this stanza:

Our heartbeats beat at the rhythm of cumbia, as mom cooks her sopes and enchiladas

It just doesn’t feel like a meal without that coke bottle

Without the gas bubbles drowning our noses and mouths

That gargling feeling that takes over our throats

Coke in glass bottles from Mexico

That gives us that taste and sensation of home

We think this possibly can’t hurt us without realizing we can’t even read the ingredients on the label

On the one hand, we could argue that youth-driven digital literacy practices are exploratory rehearsals for future navigation in online environments in health care. On the other hand, as literacy researchers, we see great value in appreciating the adolescents’ bilingual/bicultural digital practices as serious literacy achievements in their own right. In fact, by studying the digital literacy practices of immigrant adolescents, we may end up expanding our thinking about the kinds of inquiry skills we should focus on if we want to understand what “expert” digital practices look like when members of immigrant communities navigate the health care system.

### 4.4. What Unique Health Literacy Challenges and Sources of Resilience Are Experienced by Immigrant Adolescents Who Are Simultaneously Navigating the World of School, the World of English, and the World of Print Literacy

Many immigrant adolescents face a daunting task as English language learners: they must quickly develop proficiency in English while managing the complex demands of reading and writing in a variety of content areas (e.g., science, literature, and history) in a relatively short period of time. Learning is thus viewed as “double the work” for immigrant adolescents compared to native English speaking peers [56]. At particularly high risk for academic struggle are those adolescents referred to as Students with Limited or Interrupted Formal Education (SLIFE), also sometimes referred to as “unschooled youth” or “newcomers” [57,58,59]. These adolescents have experienced interruptions in their schooling in their home countries for a variety of reasons: poverty, civil wars, geographic isolation, legally sanctioned restrictions to schooling, resettlement processes that require families to move in order to verify their eligibility for assistance, and natural disasters [60].

The U.S. public school system serves an increasing number of SLIFE children, but their exact numbers are indeterminate, a function of the inconsistent approaches to assessing, classifying, and tracking this group of English language learners [59,61]. In 2007, Gunderson [62] reported that an alarming 75% of high school refugee students (who often fit the typical SLIFE profile) dropped out or disappeared from the public school system. Recent increases in SLIFE children also reflect the wave of over 175,000 unaccompanied minors who entered the U.S. from Central America from 2014 to 2017 [63].

A pervasive theme in the educational literature on SLIFE are the challenges of educating learners with serious emotional needs stemming from traumatic life experiences (e.g., war) and separation from family members. Of particular urgent concern is the adaptation struggles of unaccompanied adolescents placed into foster care who must navigate the transition into adulthood relatively quickly, and under incredibly stressful circumstances [63]. The unique socio-emotional and learning needs of SLIFE can easily get lost in the shuffle of school placement processes that inconsistently place based on age or completed years of schooling. In response, DeCapua, Smathers, and Tang [64] developed an extensive school-based interview protocol that aims to provide a safe zone for SLIFE to talk about their family circumstances, such as these excerpts:

We had to leave our home because of the war. We live in camp, my mother, my sisters, but we don’t know nothing about my father and brothers and we have nothing and we wait to come here with my uncles.

I come with my brother but my mother and other brothers stay home. I living with my father and sister and brother, but I miss my mother so much

[64] (p. 13)

Fortunately, in the public health world, there have already been numerous calls for improvements in counseling and mental health services (e.g., related to post-traumatic stress and sleep disruptions) for immigrant and refugee youth [65,66,67,68], a need recently punctuated by continued separation of refugee children from their families at the U.S.–Mexico border. However, more interdisciplinary research on the implications for immigrant adolescent health literacy is needed. As practitioners who have worked in schools, we are compelled to explore the range of health literacy tasks immigrant adolescents encounter in the context of mental health support and services provided by teachers and school counselors. More descriptive studies about the way SLIFE use their language and literacy tools to cope with stress and emotional trauma (as shown in the earlier example of the three Karen brothers) will provide additional evidence for ways that migration histories are tied to health literacy learning. Longitudinal studies will also be critical to assess changes in health literacy practices over time and the relationship to long-term adaptation outcomes.

Clearly, working with SLIFE learners presents a unique and challenging set of circumstances for teachers and school counselors; too often these professionals lack the training, time, and resources to adequately support SLIFE in schools. We do not know of any studies that have examined the communication and navigation practices of the school-based practitioners who are responsible for SLIFE well-being. Research on what works for SLIFE may improve the ways teachers, counselors, social workers, and other school staff work with SLIFE and their families. 

Another pervasive theme in the educational literature on SLIFE learners is that they learn differently from children who have print skills in their first language [69]. For example, their prior schooling experiences are anchored in spoken language which makes the transition to learning from printed texts challenging [70]. Additionally, children who have attended school in refugee camps may have been taught in different languages, depending on whether the local policy stipulated instruction in the first-language or the host-county language, further complicating the mastery of academic skills in English. Many SLIFE children need to figure out what it means to “do school” [46], which creates additional pedagogical challenges, as described by this Australian high school teacher:

… I have some Sudanese students who have had a lot of schooling who came to Australia with a very good background of English so learning is quite easy, not just the learning side of it but the rituals of school. As well I have students who have lived in refugees camps for ten years and have no background in schooling, not just the learning again, but the rituals of school, how to cope with the day to day, the system of bells and requirements… those extremes are very wide.

[71] (pp. 26–27)

This example makes us curious about the ways that SLIFE, as part of their migration and adaptive experiences, are similarly figuring out how to “do health”. More descriptive studies in the context of SLIFE’s early interactions with the U.S. health care system will give immigrant adolescents the opportunity to describe, in their own words, what learning to “do health” means when reliance on printed texts is not a productive pathway for accessing health information.

We have argued that SLIFE, a growing sector in the immigrant adolescent population, deserves more attention as a population of study in health literacy research. With more scholarship on SLIFE in literacy education in the past decade, educational researchers and teachers have had to critically examine their discourse about “illiterate” learners and question their assumptions about the role of schooling and print knowledge in literacy learning. We anticipate that research on immigrant adolescent health literacy will require a similar reckoning of assumptions about literacy that have prevailed in the health literacy field. 

### 4.5. How Do the Goals of Health Literacy Learning Differ or Overlap with Health Education Curricular Goals in Secondary School Settings?

A research agenda on immigrant adolescent health literacy needs to be able to clarify the relationship between health literacy and health education, which already has a long-established history in the U.S. public schools as a vehicle for promoting healthy outcomes among adolescents. Although a comprehensive review of U.S. health education policy and programming is beyond the scope of this paper, we caution against assuming that health literacy and health education are equivalent efforts, or that health literacy should be treated as a “school subject” in the same way that health education has been institutionalized in U.S. public schools.

The Calgary Charter acknowledges that individuals must have “some basic knowledge of science and health” and “an understanding of the health system”; however, there is no explicit recommendation as to which content areas comprise this “basic knowledge” and “understanding”. Similarly, the National Health Education Standards (NHES), most recently revised in 2007, also emphasizes the teaching of transferable skills in U.S. health education programming, partly in recognition that there was simply not enough time in the academic school year to cover potentially relevant themes at each grade level [72]. However, states still specify content areas through which schools can address these standards. For example, California specifies six content areas through which the NHES are addressed: Nutrition and Physical Activity; Growth, Development, and Sexual Health; Injury Prevention and Safety; Alcohol, Tobacco, and Other Drugs; Mental, Emotional, and Social Health; and Personal and Community Health [73]. More research is needed to specify the health literacy competencies that might be effectively reinforced in schools for immigrant adolescents at various grades, or levels of English proficiency. Such alignment would bring greater visibility to the sources of resilience and “everyday wisdom” that immigrant adolescents cultivate as health care navigators, offsetting perceptions of adolescents as at-risk youth in need of health education. 

The focus on the development of an individual learner’s skill set is prominent throughout the NHES, which contrasts with the Calgary Charter’s emphasis on health literacy as an attribute of individuals and systems. The NHES framework expects the high school learner to be able to analyze the influence of family, peers, community, media, and culture on health outcomes (Performance Indicators 2.12.1–2.12.6), but it remains unclear on how to account for the influence of those social factors on the learners’ competence. From a social perspective, however, this influence is viewed as a given. The immigrant adolescent’s health literacy and her social networks are mutually constitutive: social interactions shape health literacy, and health literacy shapes social interaction. We caution against treating health literacy learning and health education as equivalent ventures which may end up reifying assumptions that health literacy is a discrete skill set or to be treated as a “school subject” taught in schools.

As mentioned earlier, Higgins et al. makes a notable contribution, in the Canadian context, using a socio-ecological model to examine conceptual differences across health literacy, health education, and health promotion in the adolescent population [10]. Higgins et al. invites validation of their model in multilingual settings, among other contexts, which, if realized, would provide the basis for exciting partnerships between health literacy researchers and adolescent literacy practitioners in the U.S. and Canada.

## 5. Conclusions

To recap, our goal in this section is not to provide a definitive list of research directions on immigrant adolescent health literacy. Rather, we wanted to suggest some of the conceptual and practical motivations and potential benefits of doing this work (see Appendix A for a compilation of possible research directions). We already have evidence regarding the cognitive and social achievements that adolescent immigrants make as readers, writers, and thinkers exhibit in a variety of nonschool-related literacy activities, such as translating for family members or information-seeking across multiple media platforms. For those of us working in literacy education and applied linguists, we are optimistic that studies on immigrant adolescent health literacy will reveal pathways for literacy growth that may differ from what has been taken to be normative in schools. This work will affirm the significance of research on out-of-school literacies in its own right, not merely as a counter-response to research on in-school literacies. Moreover, research on immigrant adolescent health literacy will hopefully guide our pedagogical decisions as we try to help learners who may demonstrate impressive health literacy practices out of school but struggle to transfer these competencies to learning in school. 

For the health literacy field to respond meaningfully to the diverse learning needs and migration trajectories of immigrant adolescents, it must more fully reject the premise that immigrant adolescent health literacy can be reduced to a functional set of reading skills. Simply put, the health literacy field must undergo its own “social turn”. This paradigmatic shift will enable us to regard immigrant adolescent health literacy as one of many literacies adolescents learn to master, and part of a broader, evolving repertoire of communicative choices, tools for inquiry, and relationships. From the literacy as social practice perspective, we prefer to envision immigrant adolescent health literacy as a domain of social activity and adaptive change, not merely as a collection of skills and abilities. 

As an interdisciplinary research team, we know firsthand that increased collaboration between our fields of immigrant literacy and public health has reinvigorated our own health literacy research directions and sparked new thinking about how to promote health literacy among immigrant adolescents in and out of schools. We hope our commentary inspires more interdisciplinary collaborations in pursuit of empirical clarity concerning the relationship between transnationalism and the development of health literacy in the immigrant adolescent population. One concrete outcome of our own interdisciplinary collaborative efforts is a shared vocabulary, understanding, and conceptualization of how we can best support the health literacy competence of immigrant adolescents. Concepts, such as investment, give us a new vocabulary for describing the social dimensions of health literacy learning, and identify new approaches for harnessing social contexts in the design of developmentally appropriate health literacy interventions and health messaging for immigrant youth. 

With serious investment of time and funding, an interdisciplinary research agenda promises to reveal the ways immigrant adolescents learn what it means to navigate our health care system as they deploy multiple languages across a spectrum of modalities (print, oral, visual, and digital), participate in diverse social networks that include local and global peers, and make use of cultural resources in the U.S. and the home country. 

In this regard, research on immigrant adolescents can provide fresh evidence in support of health literacy as a contextualized and embedded capacity, an evolving mastery of health literacy practices as the adolescent interacts with her environment (not a discrete set of reading and writing skills learned step by step). Greater understanding of what health literacy competence looks like for this diverse population could ultimately strengthen our knowledge about more general ways that social context influences the health literacy development of all children and adults.

## Appendix A. Researching Immigrant Adolescent Health Literacy as a Socially Situated Practice: Some Exploratory Questions

### Appendix A.1. Multilingual Dimensions of Immigrant Adolescent Health Literacy

1. How does the immigrant adolescents’ knowledge of other languages, beyond English, manifest in their health literacy competence?
2. How does knowledge of other languages open or close-down routes to participation in health care encounters?
3. What is the role of oral language use in the development of immigrant adolescents’ health literacy practices?
4. To what extent do linguistic brokering encounters provide immigrant adolescents with opportunities to develop health literacy practices across functional, communicative, and critical levels?

### Appendix A.2. The Relationship between Immigrant Adolescent Health Literacy and Schooled (Academic) Literacies

1. In what ways do changes in health literacy competence reflect (or contrast with) their academic literacy competencies?
2. What skills and practices transfer across health care and school contexts?
3. How does the relationship between immigrant adolescent health literacy and schooled (academic) literacies vary for learners based on differences in print knowledge and metalinguistic awareness?

### Appendix A.3. The Link between Immigrant Adolescent Health Literacy and Digital Literacies

1. To what extent is technology enabling immigrant adolescents, despite minimal English communication skills, to “leapfrog” (Dr. Christina Zarcadoolas, personal communication, 21 March 2016) over communication barriers in health care, and expanding their ability to participate more fully in health literacy activities?
2. To what extent are the immigrant adolescent health literacy activities transnational in scope, as they use technology to interact with social groups and diverse informational resources that span geographical borders?
3. How does a focus on immigrant adolescent health literacy provide examples of immigrant adolescents’ ability to move across multiple media platforms, languages, and social contexts? How can these examples inform health literacy intervention efforts?

### Appendix A.4. The Role of Social Contexts

1. What is the range and variation of health literacy activities in which immigrant adolescents participate? How does this participation vary based on the immigrant adolescents’ family structure, level of generation, or documentation status?
2. To what extent do immigrant adolescents demonstrate an understanding of the social and institutional expectations that govern interactions in health care contexts?
3. To what extent do U.S. immigrant adolescents, as a result of their childhood experiences in health navigation and advocacy, demonstrated increased agency over their own health care choices (e.g., contraceptive use) or increased involvement in advocacy movements, such as reforms in health insurance or Deferred Action for Childhood Arrivals (DACA) policy?

### Appendix A.5. The Role of Social Networks

1. How does immigrant adolescent’s evolving health literacy competence influence their social relationships in the context of family, school, and community?
2. To what degree do their health literacy encounters reflect shifting power dynamics in their relationships to authority (parents, teachers, doctors) in their everyday lives?
3. What are strategies for harnessing immigrant adolescents’ social ties (global and local) as resources for tackling problems in health care navigation?
